# Khat – a new precipitating factor for reversible cerebral vasoconstriction syndrome: a case report

**DOI:** 10.1186/s13256-016-1155-5

**Published:** 2016-12-15

**Authors:** Harith Baharith, Amy Zarrin

**Affiliations:** 1 Department of Medicine, New York Methodist Hospital, Brooklyn, NY USA; 2 Department of Medicine, Center for Neuroscience, New York Methodist Hospital, Brooklyn, NY USA

**Keywords:** Reversible cerebral vasoconstriction syndrome, Khat, Postpartum cerebral angiopathy, Call–Fleming syndrome

## Abstract

**Background:**

Postpartum reversible cerebral vasoconstriction syndrome is one of the rare reversible cerebral vasoconstriction syndromes. The clinical presentation is usually characterized by recurrent headache, focal neurological deficit, and reversible cerebral vasoconstriction seen on cerebral angiography.

**Case presentation:**

We report a case of a 35-year-old Yemeni woman who presented with headache and focal neurological deficits that occurred 10 days after delivery, with segmental narrowing of cerebral arteries on angiography. She had significant clinical and radiological improvement on follow-up.

**Conclusions:**

The presentation of our patient’s reversible cerebral vasoconstriction syndrome is unusual as she has two possible precipitating factors. In addition to being in the postpartum state, she also has a long history of chewing khat, a vasoactive substance commonly used by immigrants from Yemen. We hope that this case report will increase awareness among physicians about the use of this plant by immigrants from the horn of Africa and Yemen.

## Background

Postpartum reversible cerebral vasoconstriction syndrome (RCVS) is one of the rare RCVSs, and commonly occurs shortly after pregnancy [1]. The syndromes have been given various names, including Call–Fleming syndrome, postpartum cerebral angiopathy, benign angiopathy of the central nervous system, thunderclap headache with reversible vasospasm, migrainous vasospasm, or migraine angiitis [1]. The syndromes are more common in women than in men and peak close to age 40 [2]. RCVS is clinically characterized by acute onset of severe headaches, with neurologic signs and symptoms, and reversible multifocal vasoconstriction of large and medium-sized cerebral arteries, which spontaneously resolves within 3 months [2]. Although the pathogenesis remains unknown, the leading hypothesis involves a disturbance in cerebral vascular tone that leads to multifocal and segmental arterial constriction [3]. RCVS may occur spontaneously or be provoked by various precipitating factors, most commonly the postpartum state or exposure to various vasoactive substances such as cocaine or serotonin reuptake inhibitors [4]. Diagnosis requires demonstration of the characteristic “string of beads” on cerebral angiography with resolution on follow-up imaging [2]. Calcium channel blockers are the most commonly used treatment [2].

We report the case of a patient with headache and focal neurological deficit that occurred 1 week after delivery, with segmental narrowing of cerebral arteries on angiography and significant clinical and radiological resolution on follow-up. The patient has a history of chewing khat (which contains various amphetamine-like substances), which we think may have provoked her symptoms.

## Case presentation

**Fig. 1:**
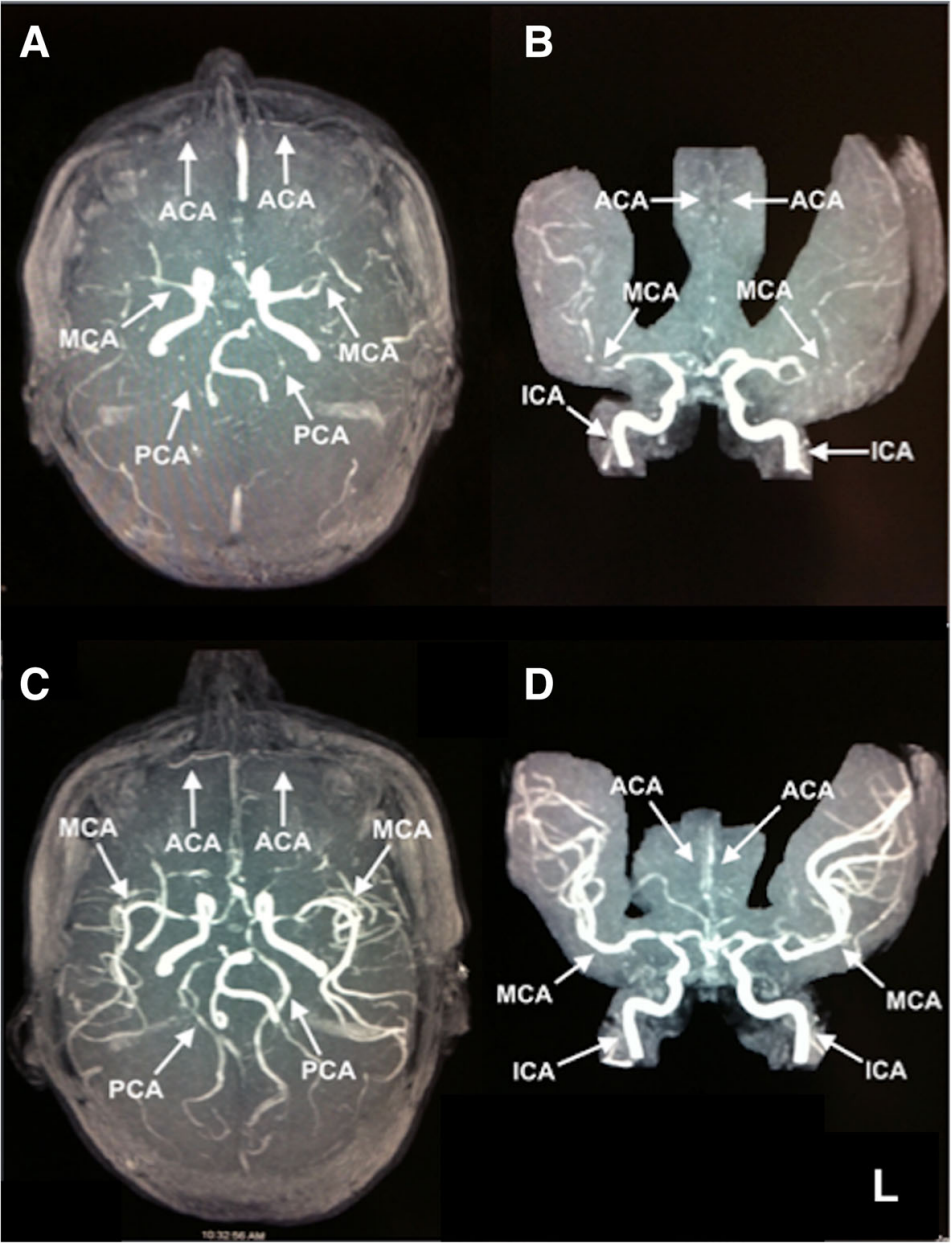
Magnetic resonance angiography on admission and follow-up. Magnetic resonance angiography of the head performed on day 1 (**a**, **b**) and day 6 (**c**, **d**) of admission. Axial (**a**, **c**) and sagittal (**b**, **d**) projections of the intracranial arteries by three-dimensional time of flight imaging. Note the decreased flow in the proximal anterior cerebral, middle cerebral, and posterior cerebral arteries bilaterally on day 1 (**a**, **b**) that significantly improves on day 6 (**c**, **d**), suggesting partial resolution of vasospasm. Internal carotid arteries were normal in both sets of images. *ACA* anterior cerebral artery, *ICA* internal carotid artery, *MCA* middle cerebral artery, *PCA* posterior cerebral artery

A 35-year-old Yemeni woman (gravida 10, para 11) with no significant past medical history but a long history of chewing khat developed throbbing headaches that started 5 days after delivery. Ten days after delivery she developed tongue heaviness and difficulty speaking with right-sided weakness, tingling, and decreased sensation and presented to our hospital’s Emergency Department. She had normal vital signs and normal cardiovascular, pulmonary, and gastrointestinal physical examinations. A neurological examination showed right facial droop, diminished power of her right upper (2/5) and lower (3/5) extremities, and decreased pain and light touch on her right hemibody. A blood workup, including complete blood count, chemistry panel, C-reactive protein, erythrocyte sedimentation rate, and autoimmune studies that included antinuclear antibodies (ANA), double-stranded DNA (dsDNA), rheumatoid factor, proteinase 3, myeloperoxidase, SS-A, SS-B, Smith, ribonucleoprotein (RNP), and Jo-1 were all unremarkable. An antiphospholipid antibody panel (beta-2 glycoprotein, phosphatidylserine, and cardiolipin), hepatitis profile, and syphilis antibody as well as electroencephalogram and echocardiogram were all unremarkable. On admission, magnetic resonance imaging (MRI) of her brain showed an acute left frontal lobe ischemic infarct. In addition, magnetic resonance angiography (MRA) of her head showed vasoconstriction of her proximal anterior, middle, and posterior cerebral arteries (Fig. 1), and a presumptive diagnosis of postpartum RCVS was made. She was started on verapamil to help decrease vasoconstriction. A repeat head MRA at approximately 1 week showed significant improvement in the vasospasm (Fig. 1). Out-patient follow-up 3 months later revealed complete clinical resolution.

## Discussion

RCVS is a rare, poorly understood and often underdiagnosed syndrome, but recently disease recognition is increasing [5, 6]. The disorder may be short lived and reversible or neurological deficits may become persistent with poor outcome if severe vasoconstriction leads to brain ischemia or hemorrhage [7]. RCVS may occur spontaneously during the postpartum period or as a reaction to various drugs. Our patient presented 1 week after delivery and despite MRI showing a left frontal stroke, she fortunately recovered completely from a clinical standpoint. In the literature, many RCVS precipitating factors have been reported for patients not in the postpartum period, including cannabis, cocaine, serotonin reuptake inhibitors, catecholamine-secreting tumors, immunosuppressants, and many other vasoactive substances [8]. Only a few cases have been reported on the development of postpartum RCVS after the use of vasoactive drugs [9–12]. In our case, the patient presented in her tenth pregnancy; no similar postpartum complication occurred in her nine previous pregnancies. Most reported cases of postpartum RCVS occur within the first three pregnancies [4]. Therefore, we suspect that she may have an additional inciting factor: use of khat. The khat plant (*Catha edulis*) is commonly chewed in social settings in Yemen, Somalia, and Ethiopia [13, 14]. It contains cathinone, a stimulant drug similar to amphetamines, which is illegal in many Western countries, including the USA and Canada; despite that, many immigrants to these countries still use khat, as did our patient. We suspect her long history of khat usage may have precipitated her condition. We think khat plant usage needs to be recognized among Yemeni immigrants who currently live in developed countries. To the best of our knowledge, there are only a few reported cases of stroke in patients consuming khat [15, 16] and one case report on khat usage and RCVS [17].

## Conclusions

RCVS is no longer an unrecognized disease. In this case, our patient with nine uncomplicated pregnancies developed postpartum RCVS on her tenth pregnancy, which is unusual. We therefore conclude that her long history of khat usage may be causally associated with her RCVS. We want the medical community to be aware that khat is a common illicit drug used by immigrants from the horn of Africa and Yemen.
